# Understanding of medical leadership from the perspective of medical students at the start of their studies and in their final year – a qualitative focus group study

**DOI:** 10.3205/zma001876

**Published:** 2026-09-15

**Authors:** Barbara Ogurek, Sigrid Harendza

**Affiliations:** 1 Universitätsklinikum Hamburg-Eppendorf, Akademie für Bildung und Karriere, Hamburg, Germany; 2 Universitätsklinikum Hamburg-Eppendorf, III. Medizinische Klinik, Hamburg, Germany

**Keywords:** leadership, competence, medical studies, professional identity

## Abstract

**Background:**

Physicians have to take on a wide range of leadership responsibilities, management as well as leadership tasks. A purely management-orientated approach is no longer sufficient to be successful as a physician leader in the dynamic healthcare sector. This explorative, qualitative study therefore analyzed the understanding of medical leadership among students in their first-semester and in their final year.

**Methods:**

Seven group discussions were conducted with 14 first-semester and 17 final-year students, participating in homogenous cohorts. These group discussions were transcribed verbatim, analyzed using Kuckartz’ content-structuring qualitative content analysis, and compared with international frameworks.

**Results:**

A total of 514 text segments were coded which could be assigned to 12 main categories comprising 49 subcategories. The main category “Demonstrating emotional-social competence“ was the most frequently mentioned in both cohorts. Differences emerged between the cohorts regarding the perspectives on personnel development though. While first-semester students emphasized the development of the leaders themselves, final-year students discussed the development of the employees more frequently. Not all aspects of the international frameworks became visible in the students’ understanding.

**Conclusion:**

Medical students showed a relatively comprehensive understanding of medical leadership, but not all aspects of recognized frameworks were included in their understanding. Since a cohort-specific perspective on some aspects of medical leadership emerged, this varying need for development could be addressed by a longitudinal curriculum on medical leadership.

## Zusammenfassung

**Zielsetzung:**

Ärzt:innen müssen eine Vielzahl an Führungsaufgaben übernehmen, die sowohl Management- als auch Leadershipaufgaben umfassen. Eine reine Managementorientierung reicht nicht mehr aus, um im dynamischen Gesundheitswesen als ärztliche Führungskraft erfolgreich zu führen. In dieser explorativen, qualitativen Studie wurde daher das Verständnis Medizinstudierender im ersten Semester und im Praktischen Jahr (PJ) von ärztlicher Führung untersucht.

**Methodik:**

Es wurden sieben Gruppendiskussionen durchgeführt, an denen 14 Erstsemester- und 17 PJ-Studierende jeweils kohortenhomogen teilnahmen. Die Gruppendiskussionen wurden verbatim transkribiert, mittels inhaltlich-strukturierender qualitativer Inhaltsanalyse nach Kuckartz ausgewertet und mit internationalen Rahmenwerken verglichen.

**Ergebnisse:**

Es wurden 514 Textsegmente codiert, die 12 Hauptkategorien mit insgesamt 49 Subkategorien zugeordnet werden konnten. Die Hauptkategorie „Emotional-soziale Kompetenz zeigen“ wies dabei die häufigsten Nennungen in beiden Kohorten auf. Unterschiede zeigten sich zwischen den Kohorten bezüglich der Perspektive auf Personalentwicklung. Während Erstsemesterstudierende die Entwicklung der Führungskraft selbst betonten, diskutierten die PJ-Studierenden die Entwicklung der Mitarbeitenden deutlich häufiger. Es wurden nicht alle Aspekte internationaler Rahmenwerke im Verständnis der Studierenden deutlich.

**Schlussfolgerung:**

Medizinstudierende zeigten ein relativ umfassendes Verständnis ärztlicher Führung, aber es waren nicht alle Aspekte anerkannter Rahmenwerke in ihrem Verständnis enthalten. Da sich kohortenbezogen eine spezifische Perspektive auf einige Aspekte ärztlicher Führung ergab, könnte dieser unterschiedliche Entwicklungsbedarf durch ein longitudinales Curriculum zu ärztlicher Führung berücksichtigt werden.

## 1. Introduction

The discussion surrounding the substantive meaning of medical leadership has changed significantly over the time. In the 1980s, the primary focus was on having physicians take on management tasks within the frame of medical leadership [1]. The emphasis was primarily on economic incentives aimed solely at increasing efficiency [2]. In the 2020s, leadership stands besides management in the focus of medical leadership [3], [4]. In times of change and a shortage of medical specialists, leadership is intended to ensure successful patient care, particularly within a team [5]. Figure 1 (Fig. 1) illustrates the concepts of management and leadership [6] and how these are combined in German-speaking countries under the term holistic leadership. Physicians have to take on a wide range of leadership tasks [7], which include clinical, administrative and management decisions [8], [9], [10]. In addition, these are complemented by further tasks, for example supporting the digital transformation [11]. These changes in the scope of medical leadership responsibilities also contribute to the acquisition of the necessary competences. As a result, analytical and strategical thinking, decisiveness, communication skills, and change management competences become increasingly important for medical leaders [11]. The latter encompass aspects such as change management, the ability to accept criticism, a willingness of learning, and the ability to motivate and delegate [11].

This development from management to leadership becomes also evident in the changes to the CanMEDS framework for competence acquisition in medical postgraduate education in Canada [12]. In the CanMEDS roles [13], [14], the physician’s role as “*Manage*r” was still described in 2005, but has shifted to the role of the *“Leader”* in the 2015 update. In Great Britain, the Medical Leadership Competency Framework was first published in 2008 by the National Health Service (NHS) as a standalone framework that comprehensively describes medical leadership competences [15]. In its further development towards the Healthcare Leadership Model, this framework now encompasses nine dimensions of leadership in healthcare [https://www.leadershipacademy.nhs.uk/healthcare-leadership-model/]. An overview of the CanMEDS roles and the dimensions of the Healthcare Leadership Model is shown in attachment 1 . 

The National Competency Based Catalogue of Learning Objectives for Undergraduate Medicine (NKLM) already reflects in medical education the ongoing development of the physician’s leadership role. In NKLM 2015, chapter 10 described „The physician as responsible person in charge and manager” [16], whereas in section VIII.5 of Version 2.0, the heading “Leadership and management” is used [https://www.nklm.de/]. As learning objective, it is described in section VIII.5.-08 that graduates should develop holistic leadership competence [https://www.nklm.de/]. In this section, the detailed objective descriptions then list task delegation, reflection on one’s own leadership style, the assumption of leadership tasks, and knowledge of leadership styles [https://www.nklm.de/]. These objectives are primarily scheduled for the last semesters and the final year. Internationally, there are already programs for developing medical leadership competence that are integrated into the curriculum in the early semesters as well as in the advanced years of undergraduate study or on a longitudinal basis [17], [18]. To date, it has been insufficiently researched, what medical students understand by medical leadership and medical leadership competence and whether their understanding of these concepts changes over the course of their studies, even though medical leadership is already firmly established longitudinally in international medical curricula [19]. Once students’ understanding of holistic medical leadership competence has been established, opportunities for acquiring such competence can be developed based on this understanding. In particular, this study aims to examine how medical students‘ understanding of medical leadership competences relates to the international medical leadership competence frameworks. To build a basis for the development of medical leadership competence, this qualitative study therefore examined what understanding of medical leadership and medical leadership competence medical students have at the beginning and at the end of their studies, whether this understanding differs between both groups, and how it compares to international frameworks.

## 2. Methods

### 2.1. Study design and participants

Between June and October 2023, seven group discussions were conducted for an explorative survey of participants’ understanding of medical leadership competence. To maximize contrast, students were included in the study as an ad hoc sample at the beginning of their studies and in their final year. The group discussions were designed to foster an interactive dynamic in order to capture broader and more thoroughly discussed perspectives than would be possible in individual interviews. A total of 31 medical students participated, of whom 16 were male (51,6%) and 15 were female (48,4%). Students in their first-semester received the information about the project during the welcome event of the onboarding week of the undergraduate medical studies iMED at the beginning of the first semester at the medical faculty of the University of Hamburg. A total of 117 students registered for participation, of whom 36 participants were systematically selected using the interval sampling method and then invited via email. A total of approximately 50 final-year students from different tertials at the University Medical Center Hamburg Eppendorf were invited via email. Fourteen first-semester students (age: 21.9±2.5 years), divided into two group discussions with six and eight students, respectively, and 17 final-year students (age 28.7±4.1 years), divided into five group discussions with two to five students each, participated. It was ensured that the students had not previously participated in other studies on this topic. Furthermore, the iMED study program does not include any courses on medical leadership [20]. Since saturation of topics was reached with these group discussions, no additional students were invited. The study was conducted in accordance with the Declaration of Helsinki and was approved by the Ethics Committee of the Chamber of Physicians, Hamburg (PV3649). Participation in this study was voluntary. All students provided their written consent. Data was analyzed anonymously. 

### 2.2. Discussion guide and conduct of group discussions

A semi structured guide was developed for the group discussion. In addition to the introduction to the context of the study, it included a question at the beginning asking participants what first came to their mind when thinking about leadership. The two core questions addressed aspects of good holistic medical leadership behavior and attitudes considered to be the basis for good leadership behavior. These two core questions were explored in greater depth with additional questions (e.g., asking for examples or specific behavior exhibited by senior physicians). The guide is shown in attachment 2 . It was designed to have an open, exploratory character as basis, which was developed in a multi-stage process based on Erpenbeck’s understanding of competence [21]. In particular, behavior as expression of competence as well as attitudes as an overarching concept for the outermost level of the model (values, rules, norms) were integrated into the guide. The first draft was developed by BO, further refined in consultation with SH, and consolidated into the final version. The open, guided group discussions were designed to determine medical students’ understanding of holistic medical leadership in order to compare it with the understanding derived from the literature. Demographic data (age, gender) were collected in writing prior to the discussion. The group discussions were conducted within homogenous cohorts (first-semester students only, final-year students only, respectively). Six group discussions were facilitated by BO (educational scientist, PhD student), one discussion was facilitated by a colleague (sociologist, PhD), and both moderators had prior experience in moderating groups. The group discussions varied by length between 46 and 72 minutes. All group discussions were audio-recorded and transcribed verbatim using the audio transcription system F4x (Dr. Dresing & Pehl GmbH). The transcripts were double-checked by BO and, where necessary, linguistically edited and anonymized. 

### 2.3. Data analysis

The transcripts were analyzed with a content structuring qualitative content analysis according to Kuckartz [22] using the software MAXQDA 24 (Release 24.9.1). The initial text analysis was conducted in conjunction with the second revision of the transcripts. Independently of one another BO (educational scientist) and SH (physician) coded two transcripts and inductively developed the main categories. These were then revised by dialogue. After that, BO coded the remaining material and differentiated the subcategories. In a discursive process SH and BO finalized the category system and coded the final text segments into the corresponding subcategories. The frequency of mentions within the categories were determined both all group discussions combined, as well as separated by stage of study (first semester and final year). In addition, the relative number of mentions was calculated in relation to the total number of coded segments in each group. The complete documentation of the research process is presented in attachment 3 .

## 3. Results

From the 514 coded text segments, 12 main categories (see table 1 (Tab. 1)) and 49 subcategories regarding medical students’ perspective on medical leadership were developed. The definitions of the individual categories and exemplary quotes from the group discussion can be found in attachment 4 . All frequencies of mentions, as well as their distribution among first-semester students (S1) and final-year students (PJ), are presented in attachment 5 . 

The main category “Demonstrating emotional-social competence” had the most subcategories, 16, and a total of 152 mentions. This accounted for 26.4% of all mentions in the S1-group and 31.2% of all mentions in the PJ-group. The subcategory “Taking responsibility” included with statements such as *“[…] one [has to] [as a leader] start taking more and more responsibility […]. It is not enough to just be good at one’s job […].”* – (S1), behaviors or descriptions of attitudes that involved responsibility or a sense of responsibility for the team or for decisions. It was most frequently mentioned (n=30), followed by the subcategory “Showing empathy” (n=24). It included statements like *“Empathy is in any case […] the key word, and [as a leader one must] generally have the ability […] to put oneself in the other person’s shoes.”* – (S1) or descriptions of appreciation, respect, or empathy towards all the people with whom one works. The next most common subcategory was „Being open for ideas” (n=17), which reflected openness to others and to new ideas or concepts, respectively, regardless of hierarchy, academic discipline and profession. It included contributions to the discussion such as* “[…] other ideas, simply being open to others. So [that one] does not […] [just] pursue one’s own concept […] and have others carry it out, but […] that I provide some guidance, but am also open.”* – (PJ). The subcategories “maintaining friendly manners”, “being approachable”, “showing humility”, and ”showing authenticity“ were exclusively mentioned by PJ-students. The second most frequently mentioned main category was “taking on management” with 77 statements (S1: 19.7%; PJ: 12.5%), which was broken down into 7 subcategories. The subcategory “structuring tasks” (n=26) was addressed with statements like *“[…, that the leader] allocates tasks correctly, so that a controlled, organized process is created.”* – (S1) and further statements addressing behaviors and descriptions of developing structure were mentioned most frequently overall. The subcategory “organizing the timeframe for work” was mentioned only in the PJ-group, the subcategory “solving problems constructively” only in the S1-group.

“Demonstrating communication skills” (n=50; S1: 5.6%; PJ: 119%) was divided as a main category into the 2 subcategories “communicating according to the situation” (n=27), which comprised 3 further subcategories, and “shaping the manner of communication” (n=23). The main category “engaging in personnel development” (n=49; S1: 2.8%; PJ: 13.1%) was divided into 6 subcategories and was the second most frequently mentioned main category of the PJ-group. Overall, the most frequently mentioned subcategory was “developing employees individually” (n=14) with statements such as *“[…] [… the leader should] [assign] tasks within the team [in such a way] that everyone can work in a manner that meets their own needs, but always with the prospect to further development.”* – (PJ). Furthermore, there were descriptions of measures that involved providing individualized support to employees, e.g., promoting career advancement, identifying an individual’s development needs and taking appropriate action, conducting development discussions (including structured annual reviews), serving as a mentor, etc. The subcategory “instructing people” was the most frequently mentioned by the PJ-group and was not mentioned at all by the S1-group. The subcategory “promoting independence” was addressed only in the PJ-group and “developing training programs” only in the S1-group. The main category “developing oneself personally as a leader” (n=44) was divided into 5 subcategories. Differences emerged here between both groups (S1: 12.4%; PJ: 6.5%). The subcategory “using self-reflection” (n=17) was mentioned most frequently. This included descriptions of behavior that serves as a form of self-reflection as part of one’s personal development, or descriptions of reflecting on or questioning one’s own behavior, respectively, e.g., *“[…] it is important that, as a leader, […] one is also willing to critically examine [oneself].”* – (PJ). The subcategory “developing social-emotional competence further” was only mentioned in the S1-group. “dealing with mistakes” (n=40) was mentioned in both groups, relatively more frequently by the PJ-students (9.2%) than by the S1-students (5.1%), and, as a main category, did not give rise to any subcategories. The main category “using leadership styles” (n=31), which was discussed more frequently overall in the S1-discussions than among the PJ-students (S1: 11.8%; PJ: 3.0%), comprised 5 subcategories. “Applying hierarchical leadership” was mentioned most frequently (n=17). The subcategory “applying situational leadership” was mentioned only in the S1-group. “Having medical knowledge” (n=30) was mentioned in both groups (S1:7.3%; PJ: 5.1%) and, as a main category, had no subcategories. The main category “promoting interprofessional collaboration” (n= 19) included 3 subcategories and was discussed in both groups (S1: 5.6%; PJ: 2.7%). The subcategory “promoting interprofessional structures” was mentioned only in the S1-group. Both main categories, “having experiential knowledge” (n=10) and “taking on role model function” (n=7), were mentioned in both groups and had no subcategories. In the main category with the fewest mentions, “pursuing targeted strategies” (n=5), were 2 subcategories: “setting goals” and “thinking strategically”, with the latter being mentioned only in the PJ-group. 

When comparing the main categories derived from the group discussions, a total of 7 of the 12 main categories of medical leadership mentioned by the students could be assigned to 6 of the 9 NHS Healthcare Leadership dimensions (see table 2 (Tab. 2)). Furthermore, the two main categories “Having medical knowledge” and “Having experiential knowledge”, which could not be mapped to the NHS Healthcare Leadership Model dimensions, could be subsequently assigned to roles other than “leader” within the CanMEDS framework, as also shown in table 2 (Tab. 2). The three main categories “dealing with mistakes”, “using leadership styles” and “pursuing targeted strategies” could not be mapped to either of the two frameworks. 

## 4. Discussion

Of the 12 main categories into which the students’ understanding of medical leadership could be categorized, “demonstrating emotional-social competence” was the most frequently cited. The behaviors of medical leaders listed under this category are also found in the NHS Healthcare Leadership Model in the dimensions *“leading with care and engaging the team” and “engaging the team”* as essential aspects of medical leadership [https://www.leadershipacademy.nhs.uk/healthcare-leadership-model/explore-the-healthcare-leadership-model/].* “Leading with care”* includes the consideration of individual needs of the team members and the development of a safe working environment for them, while *“engaging the team”* involves the appreciation of team members’ contributions, the open dealing with these contributions, and fostering teamwork. The two main categories, “engaging in personnel development” and “developing oneself personally as a leader”, are found in the Healthcare Leadership Model under the dimension* “developing capability”*, which addresses both the leader’s personal development itself as well as the team’s professional development [https://www.leadershipacademy.nhs.uk/healthcare-leadership-model/explore-the-healthcare-leadership-model/]. 

A total of 7 of the 12 main categories of medical leadership mentioned by the students can be mapped to 6 of the 9 NHS Healthcare Leadership dimensions and thus show a high degree of alignment between the students understanding of medical leadership and this international framework [https://www.leadershipacademy.nhs.uk/healthcare-leadership-model/explore-the-healthcare-leadership-model/]. Three NHS Healthcare Leadership dimensions are not clearly reflected in the students’ understanding of medical leadership: *“Inspiring shared purpose”*, which essentially involves adhering to the NHS values and their implementation, *“evaluating information”*, which mainly involves the collection and analysis of data, and *“share the vision”*, which refers more to communication, motivation, and creating a sense of purpose through visions. The main categories identified by the students in their understanding of leadership essentially reflect aspects of transactional leadership [23], [24]; by contrast, these three dimensions, which are listed in the NHS Healthcare Leadership Model, but do not fall under the main categories, focus on aspects of transformational leadership [23], [24]. Since a correlation between leadership success and transformational leadership has been demonstrated [25], [26] and, at the same time, it has also been shown that transformational leadership can be developed through training [27], these are key findings for the future design of medical curricula on medical leadership, even though they were not mentioned by the students. The main categories “having medical knowledge” and “having experiential knowledge”, which cannot to be assigned to the NHS Healthcare Leadership Model, can be assigned to the* “medical expert”* role in the CanMEDS framework and are therefore part of general medical competence, but not of the* “leader”* role [14]. However, it has been shown though that “having medical knowledge” and “having experiential knowledge” are important for being accepted as a leader [28].

The main category “dealing with mistakes”, which could not be directly assigned to either of the two international frameworks, and the error culture it addresses, is an important aspect of patient care. It could be shown that the leader’s behavior can have a significant influence on the establishment of a *“speak-up-culture”*, which contributed to patient safety [29]. These behaviors mentioned by the students thus represent an important aspect of leadership competence, as they also embody an aspect of assuming responsibility in the sense of transformational leadership and role modeling. Furthermore, it could be shown that this aspect does not yet play a central role in practical training, and that experiences students gain in this regard, for example, during simulation trainings, are viewed as new and particularly relevant [30]. This could also be one reason that “Dealing with mistakes” is mentioned considerably more often by PJ-students than by S1-students. For this reason, and given its contribution to patient safety, this aspect should be taken into account in curriculum development despite its only implicit connection to the frameworks. The main category “pursuing targeted strategies” consists of “setting goals” and “thinking strategically”. The subcategory “setting goals” corresponds in content to a subaspect of *“communication”* of the Four Cs of the physician leadership model [31], in which the four Cs stand for character, competence, caring and communication. “Setting goals” or leading by example, respectively, is also a component of transformational leadership, in which leaders contribute to employees' intrinsic motivation by defining shared goals and visions, thereby also fostering their personal development [23], [25]. “Thinking strategically” as a subcategory, cannot be attributed to any specific leadership theory, but it was identified as an overarching aspect of medical leadership competence and deemed relevant [28]. The use of different leadership styles could be shown among medical leaders [32], and also in the main category “using leadership styles”, students mention different leadership styles without consistently or clearly articulating a clear understanding providing of their effects. Different effects have been described for these styles, and they draw on various theoretical models [33], [34]. The subcategory “applying motivating leadership”, for example, encompasses several leadership theories, such as servant leadership [35] and transformational leadership [23], which can have positive effects on employee motivation [35], [36]. The laissez-faire leadership style, not mentioned by the students, would have a negative impact on leadership success [23]. It is therefore considered favorable that this style is not included in the students’ understanding of medical leadership. 

The first-semester students mentioned second most frequently the main category “taking on management”, for the final-year students this was the third most frequent mention. This indicates that management is viewed as an important part of holistic leadership in both groups, which is congruent with the view that management and leadership are needed for holistic leadership [6]. The PJ- students second most frequently addressed the main category “engaging in personnel development” in their understanding of leadership, while this ranked only ninth in the list of main categories for S1-students. S1-students, on the other hand, mention third most frequently “developing oneself personally as a leader”, whereas this was only the sixth most frequent main category for PJ-students. Interestingly, these two main categories are found in the dimension *“developing capability”* of the Healthcare Leadership Model [https://www.leadershipacademy.nhs.uk/healthcare-leadership-model/explore-the-healthcare-leadership-model/], however, they represent different developmental perspectives. Since PJ-students have observed that physicians suffer from a lack of time and are overburdened with routine tasks [37], they likely view staff development as a particularly important leadership task for physicians. Therefore, when developing a curriculum, this should be taken into account toward the end of the program. The S1-students probably put at the beginning of their medical studies a particularly strong focus on personal development and therefore also view leadership as a competence in which they can continue to grow, for example, through courses [38], [39]. Therefore, the focus on self-reflection as an important basis for medical leadership should be established right from the start of the studies and can also serve as an important cross-disciplinary foundation for the students, even if, at that stage of their studies, they are not yet able to assume medical leadership responsibilities in the strict sense. In contrast, the main category “demonstrating communication skills”, as a subaspect of medical leadership competence, ranks considerably higher in our study among PJ-students with rank 4 than among S1-students (rank 7). Presumably, PJ-students have become aware of the importance of communication during their clinical training. They also do not feel well prepared for their professional careers, including the corresponding leadership responsibilities, with respect to communication [40], [41] and would like to see more opportunities in the areas of communication, career planning, and leadership competence [40]. Perhaps this could be established as an additional program in the final year during the clinical rotation, so that the communication experiences gained during the final year can be further reflected upon through learning opportunities. 

Limitations of our study are the different group sizes in the discussions and the voluntary participation of the students. These could have led to a selection of students who were particularly interested in the topic of leadership. Furthermore, due to the number of groups and their sizes, individual participants who spoke extensively in certain groups may have influenced the frequencies of mentions used as the basis for the analysis, even if this was not immediately apparent in the dataset. An additional limitation is that one group discussion was led by a different moderator than the rest of the discussions, however, the moderator did not have a dialogical part in the conversation. A strength of our study is the intensive, non-hierarchical exchange among the participants within their respective semester groups, whereby the greatest possible difference in experience to contrast perspectives on medical leadership was achieved by including S1- and PJ-students. This study demonstrated for the first time which understanding medical students have of medical leadership. They consider emotional-social aspects of medical leadership as very central. When considering competence development as part of medical leadership, S1-students focus more on the development of the leaders themselves, while PJ-students place the emphasis on personnel development. 

## 5. Conclusion

Medical students perceive the different aspects of medical leadership to varying degrees. Moreover, differences were found between S1-students and PJ-students in their perception of several aspects of medical leadership. Although medical students have a fairly comprehensive understanding of medical leadership, not all aspects of recognized frameworks are reflected in that understanding. The varying degrees of emphasis on medical leadership aspects and the finding that, depending on study progress, students place different emphasis on certain aspects of medical leadership could serve to define learning objectives in greater detail and to develop a longitudinal curriculum on medical leadership tailored to students’ needs. Furthermore, given their relevance to leadership success, aspects of transformational leadership that were not included in the students’ understanding should be incorporated into a curriculum on medical leadership. Using the findings from this study and drawing on internationally established longitudinal curricula on medical leadership, specific learning objectives addressing the various aspects of medical leadership could be developed to match the students’ level of development and implemented using appropriate teaching formats. Such a curriculum could initially be piloted and evaluated as a required elective course. The acquired competences could be compared with those of students who did not complete a medical leadership curriculum in order to assess competence acquisition and enable continuous improvement.

## Abbreviations

- CanMEDS: Canadian Medical Education Directives for Specialists
- MLCF: Medical Leadership Competency Framework
- NHS: National Health Service
- NKLM: Competency Based Catalogue of Learning Objectives for Undergraduate Medicine
- PJ: final-year
- S1: First-semester students

## Acknowledgements

We would like to thank Dr. Sarah Prediger for the moderation of one group and all students for their participation in the group discussions.

## Notes

### Ethics approval

This study was performed in accordance with the Declaration of Helsinki. The Ethics Committee of the Chamber of Physicians, Hamburg, approved this project (PV3649). The participation in this study was voluntary. Written consent forms have been obtained.

### Author’s ORCID

Sigrid Harendza: [0000-0002-7920-8431]

## Competing interests

The authors declare that they have no competing interests.

## Supplementary Material

CanMEDS roles and Healthcare Leadership Model dimensions

Discussion guide

COREQ Checklist

Description of category system

Frequencies of coded segments per category

## Figures and Tables

**Table 1:**
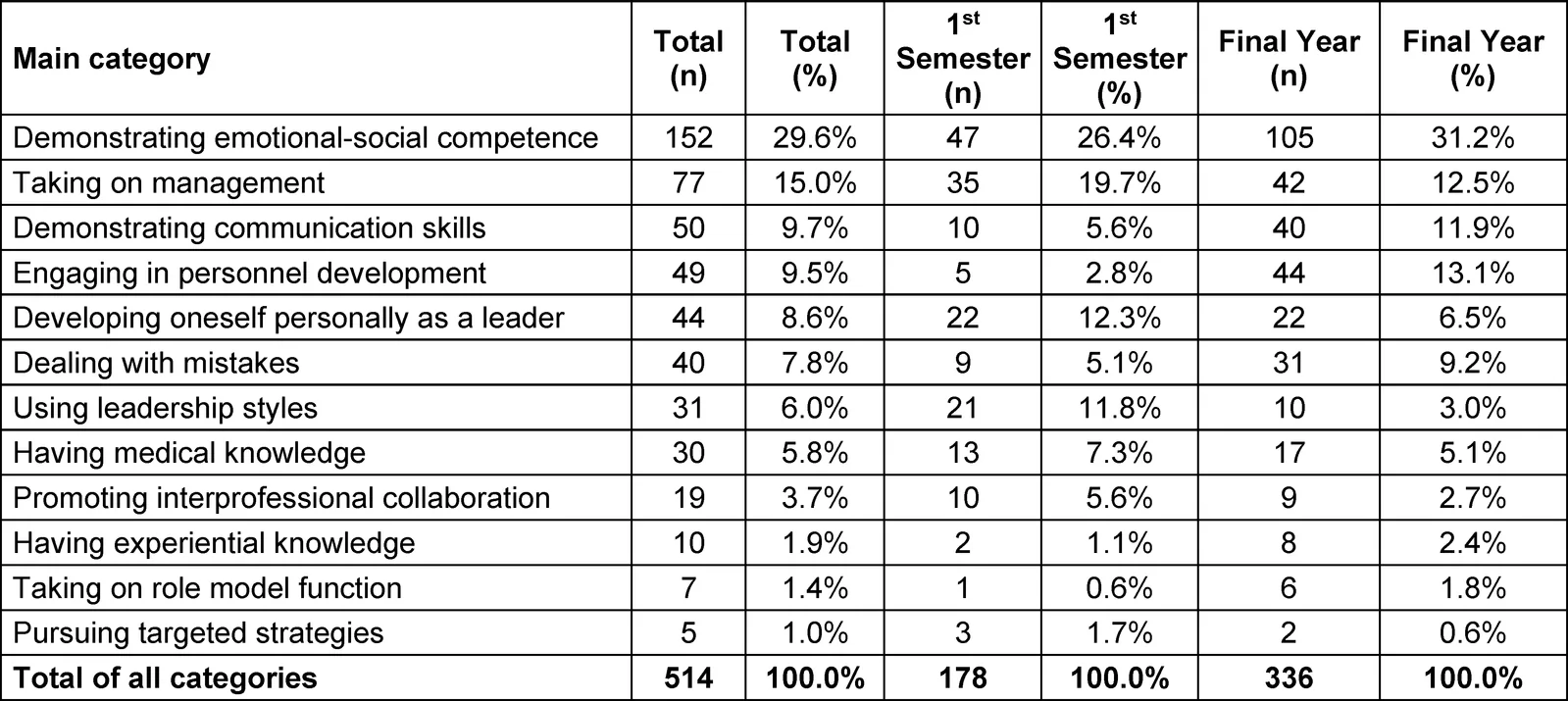
Sum of coded segments per main category

**Table 2:**
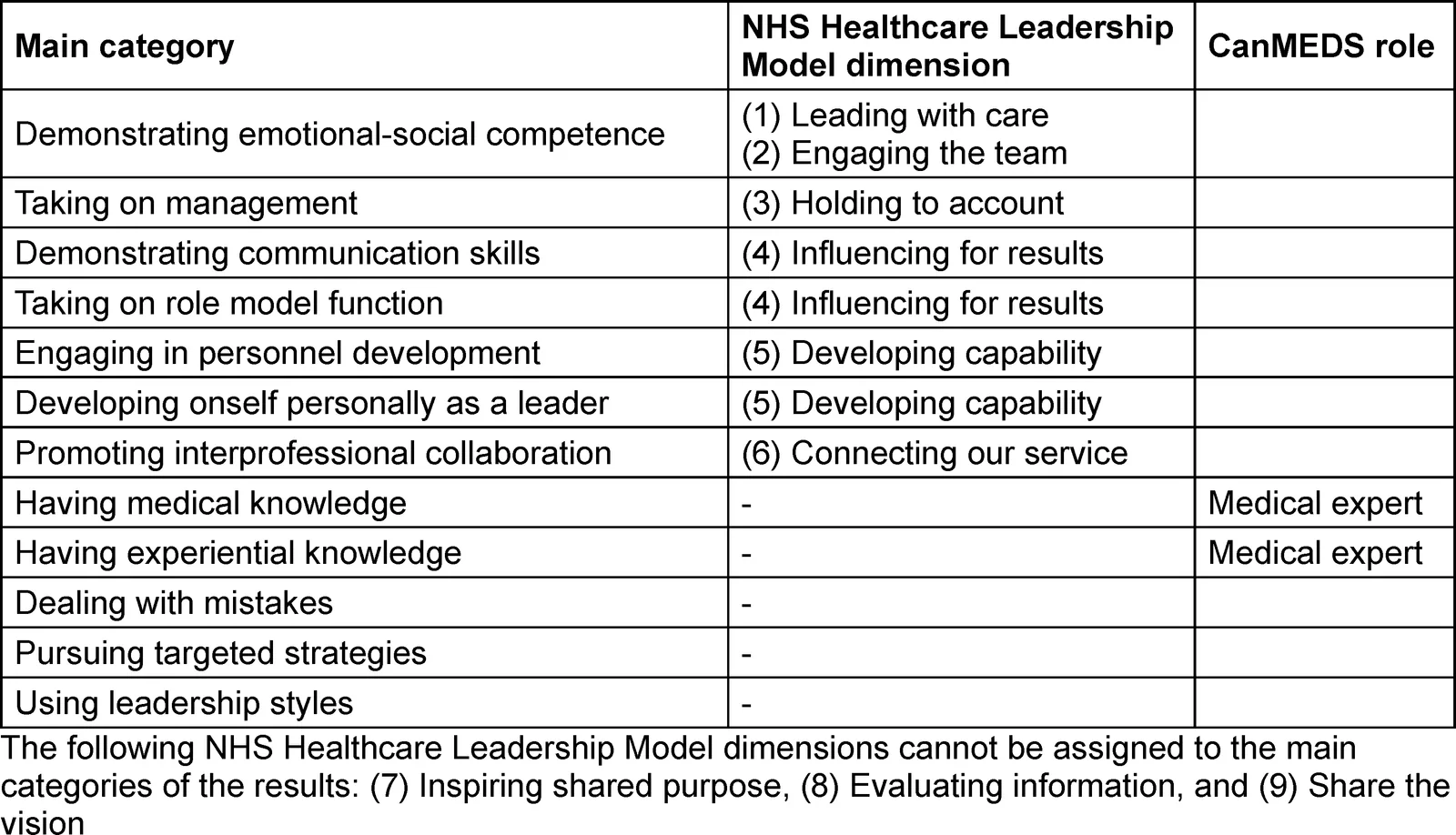
Step-by-step allocation of the main categories to the NHS Healthcare Leadership Model dimensions and the CanMEDS roles

**Figure 1:**
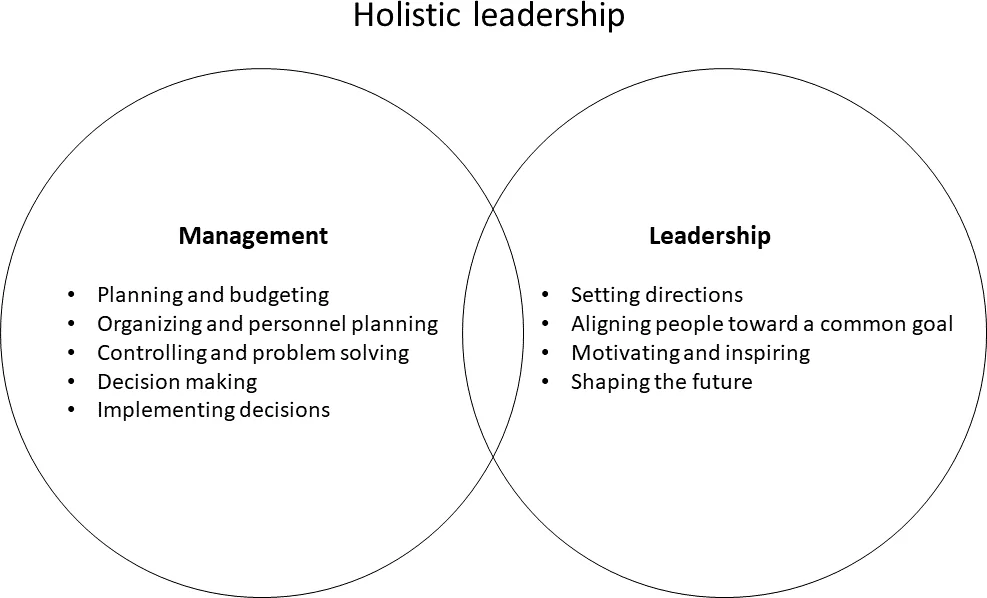
Holistic leadership
