# Supplementary material for: Understanding of medical leadership from the perspective of medical students at the start of their studies and in their final year – a qualitative focus group study
Source: GMS J Med Educ. 2026 Sep 15;43(6):Doc82. doi: 10.3205/zma001876 (PMC13617323; doi:10.3205/zma001876)
Supplement: Supplementary file 1 [file JME-43-82-s-001.pdf]

Attachment 1: CanMEDS roles and Healthcare Leadership Model dimensions

CanMEDS	Healthcare Leadership Model
Medical Expert	Leading with care
Communicator	Engaging the team
Collaborator	Holding to account
Leader	Influencing for results
Health Advocate	Developing capability
Scholar	Connecting our service
Professional	Inspiring shared purpose
	Evaluating information
	Share the vision

List of CanMEDS roles [https://www.royalcollege.ca/content/dam/document/standards-and-accreditation/2015-canmeds-framework-reduced-e.pdf] and Healthcare Leadership Model dimensions [https://www.leadershipacademy.nhs.uk/wp-content/uploads/2012/11/NHSLeadership-Framework-LeadershipFramework.pdf]

Attachment 1 to Ogurek B, Harendza S. *Understanding of medical leadership from the perspective of medical students at the start of their studies and in their final year – a qualitative focus group study*. GMS J Med Educ. 2026;43(6):Doc82. DOI: 10.3205/zma001876
