# Supplementary material for: Understanding of medical leadership from the perspective of medical students at the start of their studies and in their final year – a qualitative focus group study
Source: GMS J Med Educ. 2026 Sep 15;43(6):Doc82. doi: 10.3205/zma001876 (PMC13617323; doi:10.3205/zma001876)
Supplement: Supplementary file 2 [file JME-43-82-s-002.pdf]

Attachment 2: Discussion guide

Welcome and self-introduction. Organizational framework and data protection.

Medical leadership and leadership competences play an important role in the medical profession. I would therefore like to ask you to actively contribute your personal views on this topic to the discussion. Please feel free to express your opinion at any time; there is NO right or wrong answer.

I will guide you through the conversation and moderate the discussion.

So that we all know how to address you, please briefly introduce yourself by stating your name. All personal data will be anonymized during transcription.

Introductory question and core questions:

1. What is the very first thing that comes to mind when you think of leadership?

2. Let’s take a closer look at the medical field: Imagine, quite concretely, a senior physician who would be the perfect medical leader in your eyes. How would this person behave in a way that would make you say: THAT is a GOOD leader?

3. Imagine, quite specifically, a senior physician who, in your eyes is perfect. What kind of attitudes does this senior physician have?

Further questions:

4. We have discussed what you mean by leadership among physicians. This raises the question: How does someone become a good medical leader, a good senior physician, respectively?

5. If we summarize the discussion thus far. What do you, in summary, understand by medical leadership? From your point of view, what characterizes medical leadership?

6. During your undergraduate education/clinical rotations/final year, where have you had the opportunity to engage with the topic of medical leadership, or to develop leadership competences? (OR—for 1st-semester students: Where, in your professional journey so far, have you encountered the topic of medical leadership? To what extent have you already had the opportunity to develop leadership?)

7. Did you give any thought to the topic of medical leadership or the leadership responsibilities of a physician before you started your studies? If so, how did you become aware of the subject?
