# Supplementary material for: Understanding of medical leadership from the perspective of medical students at the start of their studies and in their final year – a qualitative focus group study
Source: GMS J Med Educ. 2026 Sep 15;43(6):Doc82. doi: 10.3205/zma001876 (PMC13617323; doi:10.3205/zma001876)
Supplement: Supplementary file 3 [file JME-43-82-s-003.pdf]

## Attachment 3: COREQ Checklist

| No. | Item | Description |
|---|---|---|
| **Domain 1: Research team and reflexivity** | | |
| | **Personal Characteristics** | |
| 1 | Interviewer/ facilitator | Section 2.2 |
| 2 | Credentials | Section 2.2 |
| 3 | Occupation | Research assistant |
| 4 | Gender | Both female |
| 5 | Experience and training | Section 2.2 |
| | **Relationship with participants** | |
| 6 | Relationship established | No prior relationship with the students |
| 7 | Participant knowledge of the researchers | Personal introduction as part of the moderation, PhD thesis topic, project topic |
| 8 | Interviewer characteristics | Reasons for the research project, independence |
| **Domain 2: Study design** | | |
| | **Theoretical framework** | |
| 9 | Methodological orientation and theory | Section 2.1 |
| | **Participant selection** | |
| 10 | Sampling | Section 2.1 |
| 11 | Method of approach | Section 2.1 |
| 12 | Sample size | Section 2.1 |
| 13 | Non-participation | Section 2.1, reasons unknown, voluntary participation |
| | **Setting** | |
| 14 | Setting of data collection | Group situation |
| 15 | Presence of nonparticipants | None |
| 16 | Description of sample | Section 2.1 |

| | | |
|---|---|---|
| | **Data collection** | |
| 17 | Interview guide | Attachment 2 |
| 18 | Repeat interviews | None |
| 19 | Audio/visual recording | Section 2.2; audiography |
| 20 | Field notes | Section 2.1; cohort-based implementation |
| 21 | Duration | Section 2.2 |
| 22 | Data saturation | Achieved when no new topics were raised in the discussions; not explicitly mentioned in the manuscript |
| 23 | Transcripts returned | Transcripts were not returned to participants |
| **Domain 3: Analysis and findings** | | |
| | **Data analysis** | |
| 24 | Number of data coders | Section 2.3 |
| 25 | Description of the coding tree | Attachment 3 |
| 26 | Derivation of themes | Section 2.3 |
| 27 | Software | Section 2.3 |
| 28 | Participant checking | None |
| | **Reporting** | |
| 29 | Quotations presented | Attachment 3 |
| 30 | Data and findings consistent | Section 3 |
| 31 | Clarity of major themes | Section 3 |
| 32 | Clarity of minor themes | No side topics included |
