# Supplementary material for: Understanding of medical leadership from the perspective of medical students at the start of their studies and in their final year – a qualitative focus group study
Source: GMS J Med Educ. 2026 Sep 15;43(6):Doc82. doi: 10.3205/zma001876 (PMC13617323; doi:10.3205/zma001876)
Supplement: Supplementary file 4 [file JME-43-82-s-004.pdf]

Attachment 4: Description of category system

Category	Description	Example
**Demonstrating emotional-social competence**		
Taking responsibility	All behaviours or descriptions of attitudes that involve or specifically describe responsibility, including verbs or adjectives such as a sense of responsibility, taking responsibility, both for the team and for decisions.	*"[…] one [has to] [as a leader] start taking more and more responsibility […]. It is not enough to just be good at one's job […]."*– (S1)
Showing empathy	Descriptions that explicitly contain the terms appreciation, respect, empathy, or their verbs or adjectives, or describe these aspects very precisely with regard to all people - employees, other people one working together with, patients.	*"Empathy is in any case […] the key word, and [as a leader one must] generally have the ability […] to put oneself in the other person's shoes."* – (S1)
Being open for ideas	Openness to others and to new ideas or concepts, regardless of hierarchy, discipline, or profession.	*"[…] other ideas, simply being open to others. So [that one] does not […] [just] pursue one's own concept […] and have others carry it out, but […] that I provide some guidance, but am also open."* – (PJ)
Demonstrating reliability	All behaviours of reliability for example in statements and agreements, provide certainty, create certainty, "with a safe pair of hands", being a person that can be trusted.	*"„[…] well, that, as a leader, one gives others as sense of security […]."* – (S1)
Demonstrating appreciation	All behaviours demonstrating appreciation or showing gratitude.	*"We need […] leadership competence […] with […] appreciation and recognition […]."* – (S1)
Maintaining friendly manners	All aspects describing to maintain friendly manners, being polite being nice towards other persons.	*"[…] a leader should] show a certain degree of friendliness […]."* – (PJ)
Demonstrating teamwork skills	Descriptions with teamwork as a behaviour or when this is made clear through descriptions.	*"[…] a senior physician [should] be able to be a team player […]."* – (PJ)
Supporting equality	Attitudes, which comprise that team members shall be treated equally, the dimensions that are relating to equality are not differentiated any further. Also included are equality aspects regarding different patient groups.	*"[…] [that as a leader] one then […] tries to make the same possible for everyone [… and] treats everyone equally […]."* – (PJ)
Maintaining professional closeness-distance	Descriptions, which mention behaviours with regard to closeness and distance explicitly, but in differentiation to accessibility, as professional closeness-distance is also related to (subjective) adequate distance.	*"[…] and with a smaller distance, which makes us [feel] closer to leaders and colleagues […]."* – (S1)<br>*"[…] but a certain amount of distance is also important, I'd say, a certain kind of relationship […]."* – (PJ)
Being approachable	Behaviours in which one is approachable or being accessible for the employees.	*"[…, that one] treats one's colleagues well and shows them 'I'm here if you need me'. […] If you […] get stuck, you can always come to me […]."* – (PJ)
Acting respectfully	To act respectfully towards other persons.	*"Well, I always associate leadership […] in the best case with the fact, that it is based, so to speak, on mutual respect […]."* – (S1)
Showing humility	Being humble in dealing with other people, which is partly also mentioned as being the opposite of being arrogant.	*"[…] when talking about the perfect senior physician, I'd say […] humility. And because the worst thing one can do as a physician is to be arrogant."* – (PJ)
Radiating confidence	Description of leading with aplomb or radiating confidence of the leading person.	*"Confident. Exactly. In any case, confident. Self-assured. Definitely. Well, that is absolutely part of it […]."* – (PJ)
Showing patience	Showing patience, especially regarding other persons.	*"One definitively needs patience with other people and also strong nerves."* – (S1)

Radiating calmness	Radiating calmness independent of the situation.	*"[…] what [the leader] must, of course, also [bring along] [is] […] the ability to react quickly and spontaneously [to] sudden events and to stay calm in such situations."* – (S1)
Showing authenticity	Reference to authentic behaviour or being authentic as a personal attitude.	*"[…, that the leader] is authentic as a person […]."* – (PJ)
**Taking on management**		
Structuring tasks	All behaviours or descriptions of structuring tasks.	*"[…, that the leader] assigns tasks properly so that a smooth, organized workflow is established."* – (S1)
Making decisions	Prioritise and making decisions regarding patient treatment that are not related to tasks structuring or team organization.	*"The first thing that comes to my mind is making decisions. […], that is […] a daily issue in a hospital that one has to make all kinds of decisions, […] when it comes to patients."* – (PJ)
Delegating tasks	All behaviours, which include task delegation, how to delegate in all steps, assign tasks and trust your employees in the process.	*"[…] delegating and assigning tasks [could] also be […] part of good leadership."* - (PJ)<br>*"[…] that one […] is willing to […] delegate tasks and then maybe also trust in the resident to handle them.*" – (PJ)
Organizing the timeframe for work	Descriptions in which the leader is expected to organise the working hours, e.g., in terms of reconciling family and career, work-life balance, reducing health risks as part of management, also in terms of resource management.	*"[…] at hospitals […], I think, work-life balance is [important]. Well, especially right now, and […] at university hospitals, a lot is expected from us, and then there is really hardly any room […] to have kids or take parental leave or be allowed to work part time and things like that."* – (PJ)
Managing resources	Planning and managing tasks that are focused on general resource management, including human resource management and which are not explicitly pointed out as organizing the timeframe of work.	*"[… it is part of a leader's tasks to] also manage resources, well, you can count in all employees."* – (S1)
Ensuring overview	Having an overview about the team, which is described as a part of management.	*„I don't believe there can really be a good team without a good leader who somehow keeps an eye on the big picture."* – (S1)
Solving problems constructively	Behaviours that include a constructive approach to problem solving.	*"I believe, a leader should also have the competence to […] view the problems they face objectively, process them. […], one should simply try to see the problem for what it is and maybe not let one's own emotions get in the way, and in the worst case, even feel personally offended by something. […] that one simply constructively tries to see and to solve the problem and make the best of the situation."* – (S1)
**Demonstrating communication skills**		
Communicating according to the situation		
Providing feedback	Behaviours that focus on providing feedback.	*"[…] that [the senior physician] gives you feedback on what you did well, what did not do so well, what you can improve.*" – (PJ)
Communicating in difficult situations	Communication in conflicts, crises, emergency situations or other situations perceived as stressful.	*"[…] speaking in front of others, communicating with others, when problems arise […]."* – (S1)
Communicating decisions	Communication in situations when decisions have to be communicated. Descriptions that explain how the communication should take place: transparent, reasoned etc. This does not mean the decision making itself, but the communication of it.	*"[…] so that one understands why [the leader] has decided like that."* – (PJ)

Shaping the manner of communication	Description of communication behaviour that explains the how to communicate, like the tone or volume.	*"[…] that one creates room for questions […] and a friendly tone. […] that the conversation is at a normal volume and […] nobody is yelling […]."* – (PJ)
**Engaging in personnel development**		
Developing employees individually	Description of measures that include the individual promotion of employees, e.g., career development, identifying development needs of individuals and initiating the relevant measures, having development talks (also as structured annual reviews), act as mentor etc.	*"[…] [… the leader should] [assign] tasks within the team [in such a way] that everyone can work in a manner that meets their own needs, but always with the prospect to further development."*– (PJ).
Instructing people	Description of behaviour, which serves the purpose of guiding employees like: Demonstrating and explaining, practical support for guidance with tasks	*"[…it is the leader's task] to provide good guidance to coworkers […] or to students […]."*– (PJ)
Imparting knowledge	Description of behaviour, which serves the purpose of imparting, passing on or teaching knowledge.	*"[…] in the transfer of knowledge, because […] the senior physician [as a leader] also contributes a great deal to the training."* – (PJ)
Promoting independence	Description of behaviour, which serves the purpose of promoting independence and develop independent work of employees.	*"[The leader must have] a sense of […] where someone can try things out or work independently, where they might not be able to, and where they need support."* – (PJ)
Enabling medical team development	Description of measures of the leader, which include promoting the team, team development and promoting cooperation within the team.	*"[…] that the ability of individual team members to work as part of a team […] is also fostered in order to strengthen this collaboration […]."* – (PJ)
Developing training programs	Description of measures, which include training programs to develop employees.	*"[…] how do I [as a leader] set up any kind of training programs."* – (S1)
**Developing oneself personally as a leader**		
Using self-reflection	Behaviour that aims at self-reflection as part of one's own development or descriptions to think about one's own behaviour, question oneself, to talk with the aim of developing oneself.	*"[…] it is important that, as a leader, […] one is also willing to critically examine [oneself]."* – (PJ)
Taking feedback from others	Accepting (external) feedback from e.g. colleagues regardless of their profession, career stage and experience. In this context the ability to take criticism is meant as willingness to accept negative feedback.	*"[… a leader should] [take] pleasure in criticism or [be] very open to criticism, or, […] above all, appreciate it when someone offers criticism of something."* – (S1)
Acquiring leadership knowledge	Descriptions, which aim at acquiring leadership knowledge and skills that could be developed in different ways, for example in courses.	*"[…], when one [as a leader] simply understands that when [using a certain technique], this happens with others, and that happens with a group […]. These are, after all, […] skills that a leader needs to know […]."* – (S1)
Developing medical specialty knowledge further	Descriptions, which aim at developing medical specialty knowledge further respectively keep up to date with changes to recommendations, guidelines, etc.	*"As a manager, one never stops learning; even if one has 30 years of experience, there may be new guidelines, new developments, or antibiotic resistance […]."* – (PJ)
Developing social-emotional competence further	Descriptions, which aim at developing all aspects summarized in the category emotional-social competency, but whereby it is not leadership knowledge that is captured here, but rather the competence-based behaviour (practical use).	*"[…], that one really does have that kind of emotional intelligence and that one tries to develop it further."* – (S1)
**Dealing with mistakes**	All descriptions regarding an error culture, communication of errors, review of errors, pointing out errors, including errors of both, superiors as well as employees etc.	*"[…], that the culture [of the leader] is [designed in such a way], that one says [as employee] 'something happened to me; it could have gone better. I did that wrong' […]."* – (PJ)

**Using leadership styles**		
Applying hierarchical leadership	Behaviours, which explicitly demonstrate hierarchy or descriptions of applying hierarchical leadership.	*"[…] and I am familiar with [leadership] from surgical departments that have very strict hierarchies […]." – (*S1)
Applying authoritarian leadership	Behaviours, which explicitly demonstrate authority or descriptions of applying authoritarian leadership, without judgement, positive or negative.	*"[…], because one holds a leadership role and should therefore project authority. Authority is important, of course, but it shouldn't turn into 'I now regard my authority as sacrosanct'."* – (S1)
Applying motivating leadership	Behaviours, which show that the leadership enhances the motivation of employees, e.g., transformational leadership or a description of servant leadership, a description of involvement of staff.	*"[…] if it is a good leader, in other words, that a team can be motivated and perhaps even be facilitated to perform better*." – (PJ)
Applying cooperative leadership	Behaviours, which explicitly demonstrate cooperative leadership or descriptions of applying cooperative leadership.	*"Yes, I believe it would be ideal, […] that one perhaps moves in the direction of a cooperative leadership style […]."* – (S1)
Applying situational leadership	Behaviours, which demonstrate leadership depending on situational factors respectively explicitly mentioned situational leadership.	*"[…] I'm thinking […] about a shift in leadership. […] [from] traditional, hierarchical systems [to] those on one level […], I would […] even […] say that there are quite a few levels in between. […] I believe that [how leadership should be exercised…] also depends very much on the leader and the specifics of the problem […]."* – (S1)
**Having medical knowledge**	Descriptions explicitly mentioning medical knowledge, medical research, whereby experience is not understood as knowledge.	*"[…] if one has good clinical competences, then this justifies at least to some extent that one is in this leadership position […]."* – (PJ)
**Promoting interprofessional collaboration**		
Engaging in interprofessional communication	All behaviours and attitudes, which contribute to communication in interprofessional settings.	*"[…], there needs to be a great deal of communication between the various professions […]. [During] the morning round, where […] a resident […and], also the nurses [were] present and then all patients were discussed, what was on the agenda, what still needed to be done. […] the senior physicians joined in later when the ward round began and as a result, things often [got] overlooked. And that's why I would like to see the senior physician to [be] much more present and also to interact with the different professions."* – (S1)
Demonstrating interprofessional team thinking	Behaviours and attitudes, which imply interprofessional team-thinking that patient care could only be done together.	*"Okay, so maybe the core team consists more of nursing staff and physicians, who do end up working more closely together. But even without the cleaning staff, the next patient can't be admitted to the room either. So, in the end, it really is a large team after all."* – (PJ)<br>*"[There …] also needs to be understanding for other professions, when one is in a leadership position."* – (S1)
Promoting interprofessional structures	Behaviours and attitudes, which promote structures, processes, delegation of tasks in an interprofessional team instead of only focussing on the physicians.	*"[…], that [as a leader] […] you flatten the hierarchy by delegating responsibility through the continuing education of nurses […], so that […] one ends up with a productive team."* – (S1)
**Having experiential knowledge**	Experiential knowledge, which is not medical knowledge. Experience or tenure is always pointed out concretely.	*"[…] [a person is promoted to a leadership position] […] with a great deal of experience."* – (PJ)

**Taking on role model function**	Behaviours, which demonstrate taking on role model function or being an example; although the nature of the exemplary behaviour could vary.	*"[…] and [that the leader] […] also acts [in everyday clinical practice] as a role model […], that this is simply part of the job and that one cannot sugarcoat every diagnosis and therapy."* – (PJ)<br>*"[…], at least for me, that kind of role model as a leader would be someone who manages to find a balance between professional and personal life […]." – (PJ)*
**Pursuing targeted strategies**		
Setting goals	Behaviours, which comprise the definition of or the description of objectives for the department, the team, the employees or the leader.	*"And if I do it the way [the leader] envisions it, then we will reach the goal."* – (S1)
Thinking strategically	Descriptions of strategic leadership behaviour or behaviour that is focussing on a strategic perspective.	*"And positive is when [a leader] thinks strategically."* – (PJ)

Attachment 4 to Ogurek B, Harendza S. *Understanding of medical leadership from the perspective of medical students at the start of their studies and in their final year – a qualitative focus group study*. GMS J Med Educ. 2026;43(6):Doc82. DOI: 10.3205/zma001876
