# Supplementary material for: Understanding of medical leadership from the perspective of medical students at the start of their studies and in their final year – a qualitative focus group study
Source: GMS J Med Educ. 2026 Sep 15;43(6):Doc82. doi: 10.3205/zma001876 (PMC13617323; doi:10.3205/zma001876)
Supplement: Supplementary file 5 [file JME-43-82-s-005.pdf]

Attachment 5: Frequencies of coded segments per category

Category	Total (n)	1st Semester (n)	Final year (n)
**Demonstrating emotional-social competence**			
Taking responsibility	30	18	12
Showing empathy	24	5	19
Being open for ideas	17	5	12
Demonstrating reliability	12	5	7
Demonstrating appreciation	10	4	6
Maintaining friendly manners	10	0	10
Demonstrating teamwork skills	8	2	6
Supporting equality	8	1	7
Maintaining professional closeness-distance	6	1	5
Being approachable	6	0	6
Acting respectfully	5	2	3
Showing humility	5	0	5
Radiating confidence	4	2	2
Showing patience	3	1	2
Radiating calmness	3	1	2
Showing authenticity	1	0	1
**Taking on management**			
Structuring tasks	26	13	13
Making decisions	22	6	16
Delegating tasks	12	8	4
Organizing the timeframe for work	6	0	6
Managing resources	5	3	2
Ensuring overview	3	2	1
Solving problems constructively	3	3	0
**Demonstrating communication skills**			
Communicating according to the situation			
Providing feedback	14	5	9
Communicating in difficult situations	7	3	4
Communicating decisions	6	1	5
Shaping the manner of communication	23	1	22
**Engaging in personnel development**			
Developing employees individually	14	2	12
Instructing people	13	0	13
Imparting knowledge	12	1	11
Promoting independence	6	0	6
Enabling medical team development	3	1	2
Developing training programs	1	1	0

**Developing oneself personally as a leader**			
Using self-reflection	17	5	12
Taking feedback from others	11	8	3
Acquiring leadership knowledge	8	5	3
Developing medical specialty knowledge further	5	1	4
Developing social-emotional competence further	3	3	0
**Dealing with mistakes**	**40**	**9**	**31**
**Using leadership styles**			
Applying hierarchical leadership	17	11	6
Applying authoritarian leadership	7	5	2
Applying motivating leadership	4	3	1
Applying cooperative leadership	2	1	1
Applying situational leadership	1	1	0
**Having medical knowledge**	**30**	**13**	**17**
**Promoting interprofessional collaboration**			
Engaging in interprofessional communication	9	4	5
Demonstrating interprofessional team thinking	6	2	4
Promoting interprofessional structures	4	4	0
**Having experiential knowledge**	**10**	**2**	**8**
**Taking on role model function**	**7**	**1**	**6**
**Pursuing targeted strategies**			
Setting goals	4	3	1
Thinking strategically	1	0	1
**Total of all categories**	**514**	**178**	**336**

Attachment 5 to Ogurek B, Harendza S. *Understanding of medical leadership from the perspective of medical students at the start of their studies and in their final year – a qualitative focus group study*. GMS J Med Educ. 2026;43(6):Doc82. DOI: 10.3205/zma001876
